# Evolution of More Aggressive LDL-Cholesterol Targets and Therapies for Cardiovascular Disease Prevention

**DOI:** 10.3390/jcm12237432

**Published:** 2023-11-30

**Authors:** Jeffrey E. Jones, Kevin S. Tang, Ailin Barseghian, Nathan D. Wong

**Affiliations:** Heart Disease Prevention Program, C240 Medical Sciences, Division of Cardiology, Department of Medicine, University of California, Irvine, CA 92697, USA; jeffreej@hs.uci.edu (J.E.J.); ktang16@hs.uci.edu (K.S.T.); barsegha@hs.uci.edu (A.B.)

**Keywords:** dyslipidemia, low density lipoprotein cholesterol, guidelines, cardiovascular disease

## Abstract

Over the last half-century, discussions on the exact targets for low-density lipoprotein cholesterol (LDL-C) reduction have evolved towards a more aggressive approach with lower LDL-C targets, particularly for high-risk patients with pre-existing atherosclerotic cardiovascular disease (ASCVD). A wealth of cardiovascular outcome trials have shown the efficacy of statin therapy in general, as well as the incremental impact of high-intensity statin therapy in particular. More recent trials have further demonstrated the impact of non-statin therapies, including ezetimibe, proprotein convertase subtilisin/kexin type 9 inhibitors, and, most recently, bempedoic acid, on reducing ASCVD outcomes. The availability of these and other newer therapies has prompted clinicians to strive for lower LDL-C targets to address residual ASCVD risk after statin therapy. This paper will provide an overview of the historical trends in lipid management and therapeutics and review the current state of evidence for lower LDL-C targets in clinical guidelines and recommendations.

## 1. Background

Cardiovascular disease (CVD) continues to be the leading cause of mortality in the United States and globally, accounting for about one-fourth of all deaths [1,2]. Whereas age, sex, family history, and genotype are fixed risk factors for cardiovascular disease, modifiable predictors of CVD include an unhealthy diet, hypertension, obesity, type 2 diabetes mellitus, and dyslipidemia—defined as the imbalance of lipids such as triglycerides, high-density lipoprotein cholesterol (HDL-C), and low-density lipoprotein cholesterol (LDL-C) [1,2,3,4,5,6].

Compelling data indicate that aggressive lipid-lowering therapy further reduces the risk of atherosclerotic cardiovascular disease (ASCVD) events, especially among individuals with established CVD [7]. The Cholesterol Treatment Trialists Collaboration has shown an approximately 20% reduction in the risk for cardiovascular disease events for every 1 mmol/L (approximately 40 mg/dL) reduction in LDL-C [8]. Moreover, lipid-lowering therapy is associated with reductions in cardiovascular events in populations with a high coronary event risk (≥30% risk: 22% reduction in major coronary events per mmol/L reduction in LDL-C) and individuals with diabetes mellitus (21% reduction in major vascular events per mmol/L reducing in LDL-C) [8,9,10]. Since 1988 and beginning with the Third Adult Treatment Panel of the National Cholesterol Education Program (ATP-III), there has been an evolution in guidelines calling for lower LDL-C targets, especially in higher-risk populations, which has corresponded with the publication of key clinical trials of both statin and non-statin therapies. The goal of this review is to synthesize this evolution of evidence and guidelines over the last several decades and provide a summary of the latest recommendations for aggressive lipid control in cardiovascular disease risk reduction.

## 2. LDL-C as a Predictor of CVD

The relationship between cholesterol and CVD was first elucidated as early as the mid-20th century. In 1939, Carl Muller observed that individuals with familial hypercholesterolemia had extreme arterial plaque deposition and faced a very high risk for cardiovascular death [11,12]. In 1952, John Gofman discovered that patients with a history of myocardial infarction (MI) have higher levels of low-density serum cholesterol than healthy individuals [13]. By 1977, the Framingham Heart Study had established serum LDL-C as an independent risk factor for ASCVD [14].

Cholesterol and triglycerides are insoluble molecules that must be complexed with proteins to be transported within circulation. Lipoproteins have a hydrophobic core of cholesterol and triglycerides surrounded by a hydrophilic membrane consisting of free cholesterol, phospholipids, and apolipoproteins. Plasma lipoproteins are grouped into categories based on size, lipid composition, and associated apolipoproteins. Chylomicrons, the largest lipoproteins, are formed from dietary triglycerides and metabolized by muscle and adipose tissue into chylomicron remnants which are absorbed by the liver. The liver’s endogenous pathway of cholesterol metabolism begins with the formation of very low-density lipoproteins (VLDL) which are subsequently metabolized into indeterminate-density lipoproteins (IDL) and further catabolized into LDL-C [15].

Biochemical studies link LDL-C and atherosclerosis via cholesterol penetration and retention in the arterial endothelium. LDL-C is oxidized and subsequently targeted by scavenger macrophages that become cytokine-secreting foam cells. Inflammation and atherosclerotic progression lead to the formation of intravascular plaque, which can cause myocardial ischemia and infarction [16,17,18].

The liver can initiate reverse cholesterol transport via the release of high-density lipoprotein (HDL) particles that acquire cholesterol from circulation. By absorbing excess cholesterol and returning it back to the liver, this mechanism reduces and inhibits the formation of atherosclerotic plaques [19]. Low serum HDL-C is associated with a higher risk for ASCVD; however, a very high HDL-C has not been shown to be protective against ASCVD [20,21,22].

Familial hypercholesterolemia (FH) is an autosomal dominant condition characterized by high serum cholesterol and as much as a 20-fold increased risk of ASCVD. Deleterious mutations in the LDL-C receptor itself or apolipoprotein B (apoB), the major ligand between LDL-C particles and their receptor, can predispose individuals to extremely high serum LDL-C. Additionally, gain-of-function mutations in proprotein convertase subtilisin/kexin type 9 (PCSK9), an enzyme that mediates LDL-C receptor degradation, resulting in very high LDL-C levels, are also an etiology for FH [23]. Patients with heterozygous FH tend to have LDL-C ≥ 190 mg/dL in adults or ≥160 mg/dL in children while homozygous FH often presents with LDL-C ≥ 400 mg/dL [24,25,26,27]. Heterozygous FH is relatively common in the general population (approximately 1 in 250–300) while homozygous FH is found in approximately 1 in 250,000–300,000 [24,28,29]. The ASCVD risk in individuals with FH is proportionate to the cumulative LDL-C burden, and treatment options consist of lipid-lowering pharmacotherapies, including statins and non-statin therapies (e.g., ezetimibe, PCSK9 inhibitors, inclisiran, and evinacumab), as well as plasma apheresis [30,31,32].

Serum LDL-C levels in many adults are influenced by diet, and a diet low in fat and cholesterol is the foundation of treatment for dyslipidemia. The average American serum LDL-C in 2000 was 127.9 mg/dL and has progressively decreased to 110.5 mg/dL in 2020 [33]. Though the decrease over time is encouraging, this may reflect the better treatment of dyslipidemia rather than prevention. Studies in different human populations demonstrate that the average American LDL-C is far from optimal. Cholesterol panels of hunter–gatherer populations, such as the Hadza of Tanzania and Pacific Islanders of Pukapuka and Tokelau, show that these groups have significantly lower serum cholesterol and a lower prevalence of cardiovascular disease compared to the US population [34,35,36]. A survey of the Tsimane of Bolivia, a population with the lowest documented prevalence of atherosclerosis, revealed that individuals have an average LDL-C of 72 mg/dL [37,38].

## 3. LDL-Cholesterol-Lowering Pharmacotherapy and Cardiovascular Outcomes Benefit

Diet and lifestyle modification can often sufficiently mitigate risk in individuals at a low risk for CVD, but higher-risk individuals and those with existing ASCVD often need pharmacologic treatment [39,40,41]. There has been a wealth of pharmacologic therapies addressing reductions in total and LDL-C, now spanning nearly 75 years (Table 1). As early as the mid-1950s, the water-soluble vitamin niacin (nicotinic acid) was identified as a pharmacologic therapy for lowering cholesterol [42,43,44]. Around the same time, the bile acid sequestrant cholestyramine was developed as another LDL-C-lowering strategy, showing significant reductions in both LDL-C and cardiovascular mortality in the Lipid Research Clinics Coronary Primary Prevention Trial [45]. Fibrates such as gemfibrozil showed early promise as an additional therapy in the Helsinki Heart Study, demonstrating a significant reduction in coronary heart disease [46]. However, these results were later complicated by the FIELD and ACCORD studies, and more recently by the PROMINENT trial, which did not show the benefit of fibrate therapy alone or when added to statin therapy [47,48,49].

The advent of statins, competitive inhibitors of hydroxymethylglutaryl coenzyme A (HMG-CoA) reductase, revolutionized how clinicians treat elevated LDL-C and reduce ASCVD risk and spurred the growth of clinical lipidology. In 1987, lovastatin became the first statin approved for human use. Statin therapy quickly became the cornerstone of lipid management, and other pharmaceuticals have been approved for further reducing LDL-C. The 1994 Scandinavian Simvastatin Survival Study (4S) demonstrated a dramatic 30% mortality reduction and a 42% reduction in major coronary events from simvastatin treatment in patients with coronary heart disease [56]. Similarly, the 1996 Cholesterol and Recurrent Events (CARE) trial of secondary prevention with pravastatin found a 24% reduction in recurrent cardiovascular events in patients who had experienced a prior MI [54]. In 1998, the AFCAPS/TEXCAPS study, a primary prevention trial, showed a 37% reduction in major coronary events from lovastatin treatment in patients with average to moderately elevated LDL-C [53]. In 2001, the MIRACL study showed that more intensive statin therapy with atorvastatin (compared to pravastatin) resulted in a further reduction in cardiovascular risk, although the endpoint was highly impacted by the reduction in hospitalization for unstable angina [66]. The Treating to New Targets (TNT) trial compared high-intensity statin therapy (atorvastatin 80 mg) to moderate-intensity therapy (atorvastatin 10 mg) in patients at a high risk for ASCVD, finding that high-intensity statin therapy further reduced cardiovascular events by 22% compared to moderate-intensity therapy [55]. The 2008 JUPITER trial investigated the use of rosuvastatin in individuals with LDL-C < 130 mg/dL but elevated C-reactive protein levels (>2 mg/L) as a measure of systemic inflammation, finding a 44% reduction in vascular events and a 54% reduction in MI compared to the control [57]. The Cholesterol Treatment Trialists’ meta-analysis of 27 randomized controlled trials of statin therapy further supported the value of statin therapy in safely reducing the risk of MI, coronary death, ischemic stroke, and coronary revascularization in a wide range of patients [8].

Ezetimibe, an inhibitor of the NPC1L1 cholesterol transporter, was approved by the FDA in 2004 as another agent for dyslipidemia. The IMPROVE-IT trial demonstrated that ezetimibe-simvastatin therapy further reduces LDL-C and adverse cardiovascular outcomes compared to statin monotherapy [58]. However, this trial was carried out in select very-high-risk ASCVD patients with an acute coronary syndrome (ACS) in the past 10 days and took 7 years to show only a modest (despite being statistically significant) benefit of a 6% relative risk reduction in the primary endpoint [58].

The advent of the proprotein convertase subtilisin/kexin type 9 (PCSK9) inhibitors has dramatically impacted the lipid therapy landscape in recent years. They provide an additional 50–60% LDL-C reduction beyond statin therapy, allowing for the achievement of LDL-C levels often below 30 mg/dL and, in some patients, below 10 mg/dL, lower than what has previously been seen from existing therapies. In 2015, alirocumab, the first FDA-approved monoclonal antibody inhibitor of PCSK9, was approved as another treatment for hyperlipidemia [61,67]. This was followed by the approval of evolocumab, whose effects on atherosclerosis were first demonstrated by the GLAGOV trial, showing that, in patients with angiographic coronary disease treated with statins, individuals taking the PCSK9 inhibitor evolocumab had significantly decreased LDL-C and a reduction in plaque atheroma volume compared to those on the placebo [68]. A reduction in ASCVD outcomes from PCSK9 inhibitor therapy was first demonstrated by the FOURIER trial, showing a 15% relative risk reduction with evolocumab-treated patients achieving median LDL-C levels of 30 mg/dL [62]. This was then followed by the ODYSSEY Outcomes trial, also showing a 15% risk reduction from treatment with alirocumab.

In 2020, bempedoic acid, an inhibitor of adenosine triphosphate citrate lyase, received approval as another lipid-lowering drug. This therapy lowers LDL-C by 15–20% as a monotherapy and by approximately 35% in a fixed-dose combination therapy with ezetimibe. In the recently reported CLEAR OUTCOMES trial, bempedoic acid reduced LDL-C by an average of 21.1% compared to the placebo in patients who were unable or unwilling to take statins owing to unacceptable adverse effects. The trial demonstrated a relative risk reduction of 13% in the primary endpoint (death from cardiovascular causes, nonfatal MI, nonfatal stroke, or coronary revascularization) in those taking bempedoic acid compared to the placebo in the overall trial population, but with a more striking 30% reduction in risk demonstrated among the primary prevention subgroup [63,69].

One year later, the small-interfering RNA inclisiran was approved for individuals who require additional LDL-C lowering. In Phase 3 clinical trials, inclisiran was associated with an approximately 50% time-averaged reduction in LDL-C [65]. Of interest, a recent analysis of 3655 patients followed for 18 months in a phase III trial of incisliran showed the therapy to be associated with a 26% risk reduction in major adverse cardiovascular events [70]. Ongoing cardiovascular outcome trials of inclisiran in both the high-risk primary and secondary prevention populations will be important for demonstrating the clinical benefit of this therapy beyond statin therapy.

## 4. Early Years of Cholesterol Treatment Guidelines

The first set of cholesterol treatment guidelines from the National Cholesterol Education Program was released in 1988. The Adult Treatment Panel (ATP) I guidelines focused on adults aged 20 years and over. Individuals with high (≥240 mg/dL) or borderline-high (200–239 mg/dL) total cholesterol with definite coronary heart disease (CHD) or at least two risk factors (male sex, family history of premature CHD, cigarette smoking, hypertension, low HDL-C, diabetes mellitus, definite cerebrovascular or peripheral vascular disease, or severe obesity) were recommended to undergo lipoprotein analysis that included LDL-C calculation [71]. At the time, primary prevention solely involved dietary therapy. If individuals continued to have LDL-C ≥ 130 mg/dL after 6 months, pharmacologic intervention with bile acid sequestrants and/or nicotinic acid was recommended. Statins were not yet recommended since their impact on CV death and the long-term safety profile had not yet been established.

The 1993 ATP II recommendations suggested HDL-C screening in all individuals, not just those with high total cholesterol. Like ATP I, these guidelines stratified patients based on total cholesterol and LDL-C. However, the LDL-C treatment goal in those with CHD was decreased from <130 mg/dL to <100 mg/dL. The risk factors for CHD were slightly changed to include men > 45 years, women > 55 years, women who had undergone premature menopause without estrogen replacement therapy, and HDL-C < 35 mg/dL. Statins (lovastatin, pravastatin, simvastatin, atorvastatin, rosuvastatin, and pitavastatin) were finally added to the list of effective pharmacologic treatments, but caution in young individuals was recommended due to the lack of long-term safety data [72].

The 2001 ATP III guidelines recommended that clinicians focus on LDL-C as the primary target for therapy [73]. These new guidelines used Framingham algorithms for the prediction of 10-year absolute CHD risk to stratify patients for treatment goals and recommended a full lipid panel be performed instead of just total cholesterol and HDL-C. The LDL-C treatment target for individuals with CHD was maintained at <100 mg/dL, but individuals with diabetes, peripheral artery disease, or a ≥20% 10-year risk of CHD were also recommended for treatment. These guidelines also recognized statins for dyslipidemia treatment given their growing evidence of benefit. The European Society of Cardiology (ESC) and the European Atherosclerosis Society (EAS) released similar guidelines in 2003, recommending a treatment target of <100 mg/dL for patients with clinically established CVD [74]. In 2004, the ATP III guidelines were updated after numerous statin trials solidified statins as an effective treatment for dyslipidemia [75]. In addition to the <100 mg/dL treatment target, an optional treatment goal of LDL-C < 70 mg/dL was added for individuals with CHD plus multiple major or poorly controlled risk factors (especially diabetes) or ACS. This target of <70 mg/dL remained the clinical standard for over a decade.

The 2006 American College of Cardiology (ACC)/American Heart Association (AHA) guidelines similarly adopted an LDL-C treatment goal of <100 mg/dL for all individuals on lipid-lowering pharmacotherapy (a Class I recommendation) and an optional target of <70 mg/dL for individuals with CHD and other clinical forms of atherosclerotic disease [76]. The 2007 ESC/EAS guidelines similarly continued the LDL-C treatment target of <100 mg/dL but added an optional treatment target of <80 mg/dL, if feasible [77]. The 2011 update to these guidelines based treatment recommendations on the 10-year predicted risk from the SCORE algorithm and recommended an LDL-C treatment target of <70 mg/dL in those at the highest risk [78]. This recommendation was unchanged in the 2016 update [79].

LDL-C reduction is a key strategy in secondary ASCVD prevention [80,81,82]. While statins are the first-line pharmacotherapy in LDL-C management, many patients at risk for ASCVD who have been prescribed statin therapy have not achieved appropriate LDL-C lowering. An earlier reduction in LDL-C in patients with ACS could be an important aspect of secondary prevention, especially considering that the greatest risk for recurrent events occurs during the month following the initial event [83,84]. Studies have demonstrated that a combination therapy of a statin plus an additional lipid-lowering agent can produce immense reductions in LDL-C and longer survival for individuals at a high risk for ASCVD. The EVOPACS study demonstrated that in patients previously hospitalized for ACS with elevated LDL-C, PCSK9 inhibitor therapy evolocumab enabled 95.7% of patients to achieve the 70 mg/dL target, compared to 37.6% of placebo group patients [85]. While the study did not identify a decrease in ACS events over the 8-week timeframe, some organizations such as the Lipid Association of India (LAI) have released recommendations for intense LDL-C lowering in patients who have experienced ACS, using ezetimibe and PCSK9 inhibitors to reach the target LDL-C of 30 mg/dL [86]. The ongoing EVOLVE-MI trial of evolocumab is testing the efficacy of evolocumab if given within 10 days of hospitalization for ACS for reducing subsequent ASCVD events.

## 5. Lower and More Aggressive LDL-Cholesterol Targets

**Figure 1:**
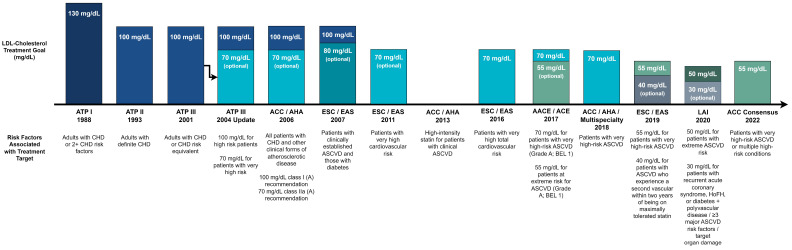
History of Cholesterol Guidelines and LDL-Cholesterol Treatment Targets. CHD, coronary heart disease; ASCVD, atherosclerotic cardiovascular disease; BEL, best evidence level; HoFH, Homozygous Familial Hypercholesterolemia; ATP, Adult Treatment Panel; ACC, American College of Cardiology; AHA, American Heart Association; ESC, European Society of Cardiology; EAS, European Atherosclerosis Society; AACE, American Association of Clinical Endocrinologists; ACE, American College of Endocrinology; LAI, Lipid Association of India.

Over the last decade, treatment targets have shifted to lower treatment and greater reduction targets for lipid-lowering therapy (Figure 1). While the 2013 ACC/AHA guideline recognized that most clinicians used LDL-C targets of <100 mg/dL and <70 mg/dL for the primary and secondary prevention of ASCVD, respectively, the guideline removed specific targets due to the lack of clinical trials testing specific LDL-C targets and focused instead on statin intensity, which prior clinical trials utilized in their design, showing the superiority of high-intensity statin over lower intensities. Higher-risk persons such as those with ASCVD, LDL-C > 190, diabetes, and multiple risk factors, or those with >20% 10-year ASCVD risk, were recommended high-intensity statin to reduce LDL-C by at least 50%, with those of an intermediate risk or diabetes without multiple risk factors recommended moderate-intensity statin designed to lower LDL-C 30–49% [87].

In 2017, the American Association of Clinical Endocrinologists and American College of Endocrinology released dyslipidemia guidelines advising that individuals at an extreme risk for ASCVD should have an LDL-C goal of <55 mg/dL [88].

In 2018, the ACC/AHA/Multispecialty cholesterol guidelines did not specify target LDL-C values for the same reasons as the 2013 guidelines but instead recommended moderate-intensity statin treatment for those of borderline (5- < 7.5%) and intermediate (7.5- < 20%) 10-year ASCVD risk (especially in the presence of additional risk enhancing factors such as a premature family history of ASCVD, persistently elevated LDL-C, metabolic syndrome, chronic kidney disease, chronic inflammatory conditions, female-specific risk enhancers such as premature menopause or pre-eclampsia, high risk race/ethnicities, or elevated lipid or other biomarkers) and high intensity statin for higher-risk primary prevention and all those with ASCVD. The “threshold” concept was also introduced as an alternative to using targets, where if the LDL-C was still at or above 70 mg/dL despite maximally tolerated statin therapy, further non-statin therapy was recommended [80]. This guideline also proposed further risk stratification of those with ASCVD into those at a very high risk (based on the presence of two or more ASCVD events or one event and multiple high-risk conditions) or not at very high risk. A more recent 2022 ACC Expert Consensus Report further reduced this threshold for the consideration of non-statin therapy to 55 mg/dL for patients with clinical ASCVD at a very high risk [82]. It recommended that ezetimibe or a PCSK9 inhibitor be considered first (because of the already published clinical outcome data at the time of writing), followed by bempedoic acid or inclisiran.

The 2019 ESC/EAS guidelines similarly recommended an LDL-C treatment goal of <55 mg/dL for very-high-risk individuals (a Class I recommendation) but lowered this even further to <40 mg/dL in patients with ASCVD who experience a second vascular event within two years (not necessarily of the same type, such as a stroke) (a Class IIb recommendation) [89].

The 2020 LAI guidelines for dyslipidemia recommended LDL-C treatment targets based upon an individual’s risk group. A treatment target of 100 mg/dL was suggested for individuals with a low or intermediate risk, and one of 70 mg/dL was suggested for individuals with a high risk. A target of 50 mg/dL was recommended for individuals at a ‘very high risk’, defined as pre-existing ASCVD, diabetes with two or more risk factors/target organ damage, or homozygous FH. For individuals with an extreme risk—those with CAD and one or more feature of a high-risk group (i.e., diabetes mellitus with zero to one other major ASCVD risk factors, CKD stage 3B or 4, coronary calcium score > 300 HU, lipoprotein (a) > 50 mg/dL or 125 nmol/L, etc.)—a treatment target of 50 mg/dL was recommended, with an optional goal of 30 mg/dL. Individuals at an extreme risk with recurrent ACS, polyvascular disease, or an additional ‘very high risk’ factor were recommended the most intense treatment target of 30 mg/dL [90].

## 6. Benefits and Risks of Very Low LDL-Cholesterol

There have been previously reported observational data suggesting an increased risk of coronary heart disease (CHD) and stroke mortality with low LDL-C levels. A recent study published in the Journal of the American Heart Association examined over fourteen thousand participants in the National Health and Nutrition Examination Survey (NHANES) over 20 years and found an increased risk of all-cause mortality (HR 1.45, 95% CI 1.10–1.93) in those with LDL-C < 70 mg/dL compared to those with LDL-C 100–129.9 mg/dL, controlling for demographic factors and comorbidities [91]. However, this observed relationship could be confounded by subclinical comorbidities not adjusted for. Moreover, clinical trials achieving low LDL-C levels, as described earlier, have not corroborated these results. However, some have shown such an association to occur in those who also have elevated high-sensitivity CRP levels > 2 mg/dL [92,93]. An earlier analysis also warned of a potential association between LDL-C < 70 mg/dL and the incidence of intracranial hemorrhage (ICH) stroke [94]; this association persists whether using LDL-C 70–99.9 mg/dL or LDL-C 100–129.9 mg/dL as the reference mark [95,96].

Notably, this association of very low LDL-C with higher rates of adverse events has not been corroborated by contemporary clinical trials. For example, the EBBINGHAUS study demonstrated no difference in effects on cognitive function with increased lipid lowering using evolocumab [97,98]; however, it is realized that we do not have such data from a longer-term follow-up. Furthermore, there are competing data suggesting that there are further cardiovascular and mortality benefits conferred with aggressive LDL-C reduction. Although the IMPROVE-IT trial found a non-significant trend towards increased hemorrhagic stroke in those treated to a lower LDL-C goal with ezetimibe and simvastatin, a subsequent 2017 analysis showed significant reductions in major adverse cardiovascular events (MACE) with LDL-C < 30 mg/dL compared to ≥70 mg/dL, without a concomitant increase in rates of neurocognitive events, hemorrhagic stroke, or non-CVD deaths [58,99]. The American Heart Association recently published a scientific statement on LDL-C lowering and the risk for dementia and hemorrhagic stroke, stating that achieving very low LDL-C does not increase the risk for hemorrhagic stroke and that the risk of a hemorrhagic stroke associated with statin therapy in patients without a history of cerebrovascular disease is small and consistently nonsignificant [100].

Notably, with lower LDL-C levels achieved in more recent trials, further benefits in ASCVD risk reduction have also been observed, further solidifying the LDL-C hypothesis. A subgroup analysis of the 2004 PROVE-IT TIMI 22 study showed that individuals who achieved LDL-C ≤ 40 mg/dL had an even greater benefit for ASCVD risk reduction (HR 0.61 compared to individuals with LDL-C 80–100 mg/dL) than those who achieved LDL-C 40–60 mg/dL (HR 0.67) [101]. The ODYSSEY OUTCOMES trial used alirocumab for extreme lipid lowering and demonstrated a 15% reduction in ASCVD events in those with a target LDL-C of 25–50 mg/dL. [61] The GLAGOV trial [68] showed greater reduction in plaque atheroma volume (regression of atherosclerosis) with the lower the LDL-C achieved down to 20 mg/dL with no evidence of a threshold effect. The FOURIER Trial compared the addition of PCSK9 inhibition with evolocumab to statin therapy with a placebo and found that the additional lowering of LDL-C to a median of 30 mg/dL resulted in a 15% reduction in the primary composite endpoint of cardiovascular death, MI, hospitalization for ACS, and coronary revascularization without a significant increase in adverse events; further reductions in cardiovascular risk were also demonstrated in this study, achieving LDL-C levels of <10 mg/dL, with no evidence of a threshold below which there was no further benefit [62]. These outcomes provide foundational evidence for the more aggressive LDL-C targets in recent international guidelines; however, more research is needed to evaluate and quantify the risks of very low LDL-C. 

## 7. Non-HDL Cholesterol and Apolipoprotein B as Secondary Treatment Targets

Statins and other lipid-lowering therapies are effective in lowering LDL-C, as well as apolipoprotein B (apoB) and non-HDL cholesterol; however, clinical trials (and thus guidelines) have all focused on LDL-C as the primary efficacy endpoint. Serum apoB correlates with the amount of circulating LDL but can also carry other atherogenic lipid particles not reflected when measuring LDL-C. Similarly, non-HDL cholesterol is a more inclusive measurement of atherogenic lipids compared to LDL-C. Analyses of major statin trials report significant decreases in apoB and non-HDL with statin use, though some suggest that statin treatments reduce LDL-C by a greater percentage than apoB and non-HDL [102,103].

Recent data have indicated that apoB and non-HDL cholesterol may be more accurate predictors of ASCVD risk compared to LDL-C in statin-treated patients [104]. One meta-analysis predicts that ASCVD risk assessment strategies centered on non-HDL cholesterol and apoB would prevent 300,000 to 500,000 more ischemic cardiac events over 10 years compared to assessment based on LDL-C [105]. The ACC, AHA, ESC, and National Lipid Association (NLA) already recognize apoB and non-HDL as risk-enhancing factors for ASCVD [78,89,90,106]. In 2015, the NLA released dyslipidemia guidelines specifying both LDL-C and non-HDL cholesterol as primary treatment targets and recommended a treatment goal of <100 mg/dL for non-HDL cholesterol, one of <70 mg/dL for LDL-C, and a secondary optional apoB target of <80 mg/dL for individuals with a very high ASCVD risk [106]. The 2016 ESC/EAS dyslipidemia guidelines similarly specified a treatment target of <100 mg/dL non-HDL cholesterol and <80 mg/dL apoB for patients with a high total cardiovascular risk as a Class IIa recommendation [79]. The 2022 ACC Expert Consensus decision pathway recommended the consideration of nonstatin therapy if non-HDL cholesterol exceeds the LDL-C treatment target by 30 mg/dL (non-HDL cholesterol ≥ 85 mg/dL for adults with clinical ASCVD at a very high risk) [82]. Whether non-HDL cholesterol or apoB eventually will replace LDL-C as the primary lipid measure for ASCVD risk remains to be seen and will depend on more robust data and the consideration of testing costs.

## 8. Conclusions

While the pathophysiology of atherosclerotic plaque formation and its relation to serum levels of LDL-C have long been known, the optimal degree of LDL-C reduction remains an important topic of consideration. While some studies have suggested an increased risk of adverse events, particularly hemorrhagic stroke with aggressive LDL-C reduction, this association has not been consistently demonstrated. Updates to several international guidelines have sourced data from numerous large trials in the last decade to inform new recommendations for lower LDL-C targets that have been demonstrated to confer a further reduction in cardiovascular morbidity and mortality. Guidelines are by nature destined for obsolescence, and emerging evidence aiding in the quantification of ideal LDL-C targets for moderate- and high-risk patients will have important clinical practice implications for years to come.

## Figures and Tables

**Table 1:** LDL-lowering Pharmacotherapies.

Drug	Year of FDA Approval	Mechanism of Action	Major Randomized Controlled Trials
Nicotinic acid (Niacin)	1950s *	Mechanism not well defined	**Coronary Drug Project**: Patients with a history of myocardial infarction on nicotinic acid had an 11% lower mortality compared to those on placebo [50]. **Atherothrombosis Intervention in Metabolic Syndrome with Low HDL/High Triglycerides: Impact on Global Health Outcomes (AIM-HIGH) and Heart Protection Study 2–Treatment of HDL to Reduce the Incidence of Vascular Events (HPS2-THRIVE)** did not demonstrate reductions in vascular events compared to statin monotherapy [51,52].
Bile acid sequestrants (Cholestyramine, Colesevelam, Colestipol)	1970s *	Increased cholesterol metabolism via bile excretion	**Lipid Research Clinics Coronary Primary Prevention Trial (LRC-CPPT)**: Colestipol reduced the risk of coronary heart disease mortality by 24% in middle-aged men with primary hypercholesterolemia [45].
Fibrates (Gemfibrozil, Fenofibrate)	1970s *	Promote receptor-mediated LDL-C clearance and increased catabolism of LDL-C	**Helsinki Heart Study**: Gemfibrozil was associated with a 34% reduction in incident coronary heart disease in middle-aged men with dyslipidemia [46]. **FIELD (Fenofibrate Event Lowering and Intervention in Diabetes) and ACCORD (Action to Control Cardiovascular Risk in Diabetes)** did not show significant reductions in cardiovascular events with fenofibrate monotherapy or in combination with other lipid-lowering medications [47,48].
Lovastatin	1987	Competitive inhibitor of HMG-CoA reductase	**Air Force/Texas Coronary Atherosclerosis Prevention Study (AFCAPS/TEXCAPS):** Lovastatin reduced the risk of major coronary events by 37% in patients with moderately elevated cholesterol [53].
Pravastatin	1991	Competitive inhibitor of HMG-CoA reductase	**Cholesterol and Recurrent Events (CARE):** Pravastatin decreased the incidence of fatal coronary events or nonfatal myocardial infarction by 24% in patients with myocardial infarction who had plasma total cholesterol levels below 240 mg/dL [54].
Atorvastatin	1996	Competitive inhibitor of HMG-CoA reductase	**Treating to New Targets (TNT)**: Intense lipid lowering with 80 mg/day atorvastatin showed a 22% relative risk reduction in cardiovascular events over treatment with 10 mg/day in patients with stable coronary heart disease [55].
Simvastatin	1998	Competitive inhibitor of HMG-CoA reductase	**Scandinavian Simvastatin Survival Study (4S)**: Simvastatin treatment was associated with a 30% reduction in death in patients with coronary heart disease [56].
Rosuvastatin	2003	Competitive inhibitor of HMG-CoA reductase	**Justification for the Use of Statins in Prevention: An Intervention Trial Evaluating Rosuvastatin (JUPITER):** Rosuvastatin decreased cardiovascular events by 44% in patients with LDL-C < 130 mg/dL but elevated C-reactive protein [57].
Ezetimibe	2004	Inhibitor of the NPC1L1 cholesterol transporter	**Improved Reduction of Outcomes: Vytorin Efficacy International Trial (IMPROVE-IT)**: Ezetimibe-simvastatin therapy provided reduction in LDL-C, with a 6% relative risk reduction in adverse cardiovascular outcomes compared to statin monotherapy [58].
Lomitapide	2012	Microsomal triglyceride transfer protein inhibitor	Phase III trials in patients with homozygous familial hypercholesterolemia on current lipid-lowering therapy demonstrate a 50% LDL-C reduction (8.7 mmol/L to 4.3 mmol/L) at 26 weeks [59].
Mipomersen	2013	Small interfering RNA inhibitor of apolipoprotein B	A randomized controlled trial of individuals with familial hypercholesterolemia on lipid-lowering therapy showed a 36% reduction in LDL-C and significant reductions in apolipoprotein B [60].
Alirocumab	2015	Monoclonal antibody inhibitor of PCSK9	**Evaluation of Cardiovascular Outcomes After an Acute Coronary Syndrome During Treatment with Alirocumab (ODYSSEY OUTCOMES)**: Alirocumab treatment resulted in a relative risk reduction of 15% for ASCVD events compared to the placebo in acute coronary syndrome patients on statin therapy [61].
Evolocumab	2015	Monoclonal antibody inhibitor of PCSK9	**Further Cardiovascular Outcomes Research with PCSK9 Inhibition in Subjects with Elevated Risk (FOURIER)**: Evolocumab treatment was associated with a relative risk reduction of 15% for ASCVD events in patients with ASCVD on statin therapy [62].
Bempedoic Acid	2020	Adenosine triphosphate-citrate lyase inhibitor	**Cholesterol Lowering via Bempedoic Acid, an ACL-Inhibiting Regimen (CLEAR OUTCOMES)**: Bempedoic acid compared to placebo given to patients with statin intolerance showed a reduction in the primary endpoint of death from cardiovascular causes, nonfatal myocardial infarction, nonfatal stroke, or coronary revascularization of 13% [63].
Evinacumab	2021	Monoclonal antibody inhibitor of angiopoietin-like protein 3	**Evinacumab for Homozygous Familial Hypercholesterolemia (ELIPSE HoFH):** Evinacumab decreases LDL-C by 49% in patients with homozygous familial hypercholesterolemia (average LDL-C 255.1 mg/dL) on a maximum background lipid-lowering therapy [64].
Inclisiran	2021	Small interfering RNA inhibitor of PCSK9	**Inclisiran for Participants with Atherosclerotic Cardiovascular Disease and Elevated Low-density Lipoprotein Cholesterol (ORION-10 and 11)**: Inclisiran reduces LDL-C by 50% in ASCVD patients on maximally tolerated statin [65]. Cardiovascular outcomes trials are ongoing.

* Date reflects approximate period of significant adoption. HMG-CoA reductase, Hydroxymethylglutaryl coenzyme A reductase; PCSK9, Proprotein convertase subtilisin/kexin type 9.

## Data Availability

This review paper did not involve creation or analysis of new data.
